# Hengli^®^ Chinese Botulinum Toxin Type A for Treatment of Patients With Overactive Bladder: A Multicenter, Prospective, Randomized, Double-Blind, Placebo-Controlled Trial

**DOI:** 10.3389/fphar.2022.840695

**Published:** 2022-02-18

**Authors:** Limin Liao, Qinggang Liu, Huiling Cong, Zhihui Xu, Enhui Li, Zhiliang Weng, Haihong Jiang, Ben Liu, Xiao Huang, Shujie Xia, Wei Wen, Juan Wu, Guowei Shi, Yang Wang, Peijun Li, Yang Yu, Zujun Fang, Jie Zheng, Ye Tian, Donghao Shang, Hanzhong Li, Zhongming Huang, Liqun Zhou, Yunxiang Xiao, Yaoguang Zhang, Jianlong Wang, Xiaodong Zhang, Peng Zhang, Dongwen Wang, Xuhui Zhang, Keji Xie, Bin Wang, Lulin Ma, Xiaojun Tian, Lijun Chen, Jinkai Dong

**Affiliations:** ^1^ Department of Urology, China Rehabilitation Research Center, School of Rehabilitation Medicine, Capital Medical University, Beijing, China; ^2^ Cheeloo College of Medicine, Shandong University, Jinan, China; ^3^ University of Health and Rehabilitation Sciences, Qingdao, China; ^4^ Department of Urology, Zhejiang Provincial People’s Hospital, Hangzhou, China; ^5^ Department of Urology, First Affiliated Hospital of Wenzhou Medical University, Wenzhou, China; ^6^ Department of Urology, First Affiliated Hospital of Zhejiang University School of Medicine, Hangzhou, China; ^7^ Department of Urology, Shanghai First People’s Hospital, Shanghai, China; ^8^ Department of Urology, Shanghai Fifth People’s Hospital, Shanghai, China; ^9^ Department of Urology, Ningxia Medical University General Hospital, Xining, China; ^10^ Department of Urology, Huashan Hospital, Fudan University, Shanghai, China; ^11^ Department of Urology, Beijing Friendship Hospital, Capital Medical University, Beijing, China; ^12^ Department of Urology, Peking Union Medical College Hospital, Chinese Academy of Medical Sciences, Beijing, China; ^13^ Department of Urology, Peking University First Hospital, Beijing, China; ^14^ Department of Urology, Beijing Hospital, Beijing, China; ^15^ Department of Urology, Beijing Chaoyang Hospital, Capital Medical University, Beijing, China; ^16^ Department of Urology, First Hospital of Shanxi Medical University, Taiyuan, China; ^17^ Department of Urology, Guangzhou First People’s Hospital, Guangzhou, China; ^18^ Department of Urology, Peking University Third Hospital, Beijing, China; ^19^ Department of Urology, Fifth Medical Center of PLA General Hospital, Beijing, China

**Keywords:** botulinum toxin type A, Hengli^®^, overactive bladder, randomized controlled trial, China

## Abstract

**Objective:** To evaluate the efficacy and safety of Hengli^®^ Chinese botulinum toxin type A (BTX-A; 100 U) in Chinese patients with overactive bladder.

**Methods:** This study was a multicenter, randomized, double-blind, placebo-controlled trial in Chinese patients who were inadequately managed with anticholinergic medications. Eligible patients were randomized 2:1 to receive intradetrusor injections of Hengli^®^ BTX-A (*n* = 144) or placebo (*n* = 72). The primary endpoint was the change in the number of daily micturition episodes at week 6 from baseline. The secondary efficacy endpoints included the average frequency of urgency and urinary incontinence (UI) episodes per day, urgency score, average micturition volume per day, OABSS, and QoL score.

**Results:** In the Hengli^®^ BTX-A group, there was a significantly greater reduction in the average number of micturition episodes per 24 h compared with the placebo group (3.28 vs. 1.43; *p* = 0.003). Moreover, there was a significantly greater improvement in the daily number of urgency episodes, micturition volume and OABSS score. An increased post-void residual urine volume, dysuria, and urinary tract infection represented adverse events (AEs) in the Hengli^®^ BTX-A group. Most AEs were mild or moderate in severity. One patient in the BTX-A group initiated clean intermittent catheterization (CIC) during treatment.

**Conclusion:** Hengli^®^ BTX-A treatment was well-tolerated and resulted in significant improvements in OAB symptoms among Chinese patients inadequately managed by anticholinergics.

**Clinical Trial Registration:** http://www.chinadrugtrials.org.cn/clinicaltrials.prosearch.dhtml, Identifier: CTR20131190.

## Introduction

As defined by the International Continence Society, overactive bladder (OAB) is characterized by symptoms of “urgency, with or without urge incontinence, usually accompanied by frequency and nocturia” (Abrams et al., 2002). OAB is a common disabling condition (Irwin et al., 2011). OAB has detrimental effects on daily activities and mental health, thus significantly reducing the quality of life (QoL) (Abrams et al., 2000; Irwin et al., 2006). According to an epidemiologic survey conducted in China, the prevalence of OAB is 23.9% in adults >40 years of age, and increases proportionally with advancing age (Chuang et al., 2019).

Anticholinergic agents are the mainstay of pharmacologic treatment for OAB (Allahdin and Oo, 2012; De Nunzio et al., 2021). Use of anticholinergic agents, however, is often restricted by insufficient efficacy and/or intolerable side effects, such as constipation and dry mouth (Benner et al., 2010; Sexton et al., 2011). Alternatively, minimally invasive options are justified in the case of inadequate symptom control, including minimally posterior tibial nerve stimulation (PTNS), sacral nerve stimulation (SNS), and intradetrusor botulinum toxin A (BTX-A) injections (Lightner et al., 2019). BTX-A is produced from *Clostridium* botulinum and inhibits the release of neurotransmitters at presynaptic nerve terminals. When administered into the detrusor muscle of the bladder, BTX-A inhibits the release of acetylcholine, thus suppressing involuntary contractions of the detrusor muscle. Additionally, it has been suggested that BTX-A suppresses afferent pathways by blocking the release of neurotransmitters, including substance P and adenosine triphosphate (ATP), and inhibiting expression of the TRPV1 and P2X3 receptors (Apostolidis et al., 2006; Leong et al., 2010).

Currently, there are several commercial brands of BTX-A available, including Botox^®^ (Allergan, Irvine, CA, United States), Dysport^®^ (Ipsen, Slough, United Kingdom), Xeomin^®^ (Merz Pharma, Frankfurt am Main, Germany) and Hengli^®^ (Lanzhou Biological Products Institute, Lanzhou, China) (Jiang et al., 2015). The efficacy and safety of Botox^®^ BTX-A for OAB has been well-established in two large, phase III placebo-controlled trials and in a long-term extension study conducted in the United States and Europe (Chapple et al., 2013; Nitti et al., 2016; Nitti et al., 2017). Since Hengli^®^ BTX-A was approved by the China Ministry of Health in 1993 as a national new drug, it has been produced and sold for more than 20 years. The trade names, Hengli^®^, Prosigne^®^, and Lantox^®^, are used in China, Brazil, and Russia, respectively. It has been concluded that Lantox^®^ and Botox^®^ have similar efficacy, safety, tolerability profiles, and equivalence of doses for the treatment of blepharospasm, hemifacial spasm, and cervical dystonia (Rieder et al., 2007; Quagliato et al., 2010); however, there are no large randomized trials to confirm the safety and efficacy of Hengli^®^ BTX-A in Chinese adults with OAB. Herein, we report a multicenter, randomized, double-blind, placebo-controlled study to evaluate the efficacy and safety of Hengli^®^ BTX-A treatment in Chinese patients with OAB.

## Patients and Methods

### Study Participants

Eligible patients were 18–75 years old with a primary diagnosis of idiopathic OAB and average ≥8 micturitions per day. The patients were not adequately managed with anticholinergic drugs (insufficient efficacy and/or intolerable side effects). Anticholinergics were maintained at the same dose in patients who were treated at the time of enrollment, while patients not treated with anticholinergics were not administered anticholinergics for the duration of the study. Patients not using clean intermittent catheterization (CIC) at baseline were required to initiate CIC if needed.

### Study Design

The key exclusion criteria included dysuria, post-void residual (PVR), urine volume >50 ml with spontaneous micturition, long-term indwelling catheterization or CIC, previous botulinum toxin treatment for any urologic condition within 6 months before screening, bladder or prostate cancer, and any urologic abnormalities or diseases, such as urinary tract infection (UTI) and urolithiasis, which may affect bladder function. The definition of UTI in this study included a positive urine culture (bacteriuria count >10^5^ colony forming cells/ml) and leukocyturia (>10 cells per high power field) without distinguishing between symptomatic and asymptomatic UTIs.

Throughout the period of April 2016 to December 2018, 17 sites across China were selected to participate in this multicenter, randomized, double-blind, placebo-controlled study. The study protocol was reviewed and approved by the NMPA of China (clinical trial ID: 2013L01406). All study sites received Ethics Committee approval before study initiation and all participants provided written informed consent before enrollment in the study. The trial was conducted in accordance with the Declaration of Helsinki and Good Clinical Practice guideline.

There was a screening period of 1 week before treatment in which patients presenting to this clinic completed a 3-day bladder diary, urinalysis, PVR measurement, and other laboratory tests, as well as relevant evaluations. The participants were recruited based on the results of the 1-week screening period. Following the 1-week screening period, all eligible patients were randomly assigned using the automated Interactive Web Response System (IWRS) to botulinum toxin A (100U, Hengli^®^; Lanzhou Biological Products, Lanzhou, China) or placebo in a 2:1 ratio. Stratification was based on whether or not anticholinergic therapy used. The study drug was injected into the detrusor layer approximately 2 mm deep with 0.5 ml per injection site and separated by 1 cm between each site. A total of 20 sites, including the bladder trigone, were injected with the special 5F injection needle. The bladder neck was avoided during injection. Patients in the placebo group received the same volume of placebo without BTX-A, and only freeze-dried adjuvant preparations, which are combinations of gelatin, sucrose, and dextran. Patients were administered local or general anesthesia. Antibiotics were administered prophylactically before injection and continued for 1–2 days afterward. The patients were evaluated 2, 6, 12, 14, 18 and 24 weeks after treatment to assess the efficacy and safety of the drug. If the frequency of micturition decreased <50% at 12 weeks compared to baseline and the patient was willing to undergo retreatment with botulinum toxin A, then there was a window for expansion. In the extended treatment phase, patients could undergo BTX-A retreatment beginning at week 12 for both groups and were followed up at weeks 14, 18, and 24 post-treatment.

### Efficacy and Safety Evaluations

A 3-day bladder diary was recorded to evaluate OAB symptoms before baseline and at each follow-up evaluation. The primary efficacy endpoint of this study was the change from baseline in the number of micturition episodes per 24 h after 6 weeks of treatment. The secondary efficacy endpoints included the change in the average number of micturition episodes per 24 h from baseline at weeks 2 and 12, as well as the change from baseline in the average frequency of urgency and urinary incontinence (UI) episodes per day, urgency score, average micturition volume per time, overactive bladder symptom score (OABSS) (Homma et al., 2006), and QoL score at weeks 2, 6, and 12 after treatment. The health-related (HR) QoL was assessed using the International Prostate Symptom Score-QoL Subscore (Chapple et al., 2009).

Adverse events (AEs), PVR, CIC were monitored throughout the trial. In patients who do not use CIC, PVR measurements were also recorded. Retention of urine was defined as a PVR ≥200 ml that required CIC. As was the case in patients with urinary retention, the decision to initiate CIC post-treatment rested primarily with the investigator’s clinical judgment. Specifically, CIC was initiated if the PVR was >200 and <350 ml and if the patient had symptoms that warranted CIC, or if the PVR was >350 ml with or without symptoms.

### Statistical Analysis

The full analysis set (FAS) population was analyzed (all randomized patients except three who did not use experimental drugs as prescribed) to determine efficacy. The safety population (all patients who received treatment, analyzed by treatment received) was analyzed to determine safety. The planned enrollment was 216 patients, including a 20% attrition rate. The study design provided 80% power to detect a between-group difference as reported in a previous study in change from baseline of 1.43 micturition times per 24 h at week 6, assuming a common standard deviation of 3 micturition times (Chapple et al., 2013).

Comparisons of numerical variables between groups were done using t-tests and Wilcoxon’s test. An analysis of categorical variables between two groups was done using a chi-squared test and Fisher’s exact test. Analysis of continuous variables and categorical variables within the treatment groups was performed using paired t-tests and Wilcoxon signed-rank tests. A significance level of 0.05 was used for all statistical tests. For data with a normal or non-normal distribution, the mean ± standard deviation or median (interquartile range) was used. Statistical calculations were performed using SAS (version 9.2; SAS Institute Inc., Cary, NC, United States).

## Results

### Patient Demographics and Disease Characteristics

**FIGURE 1:**
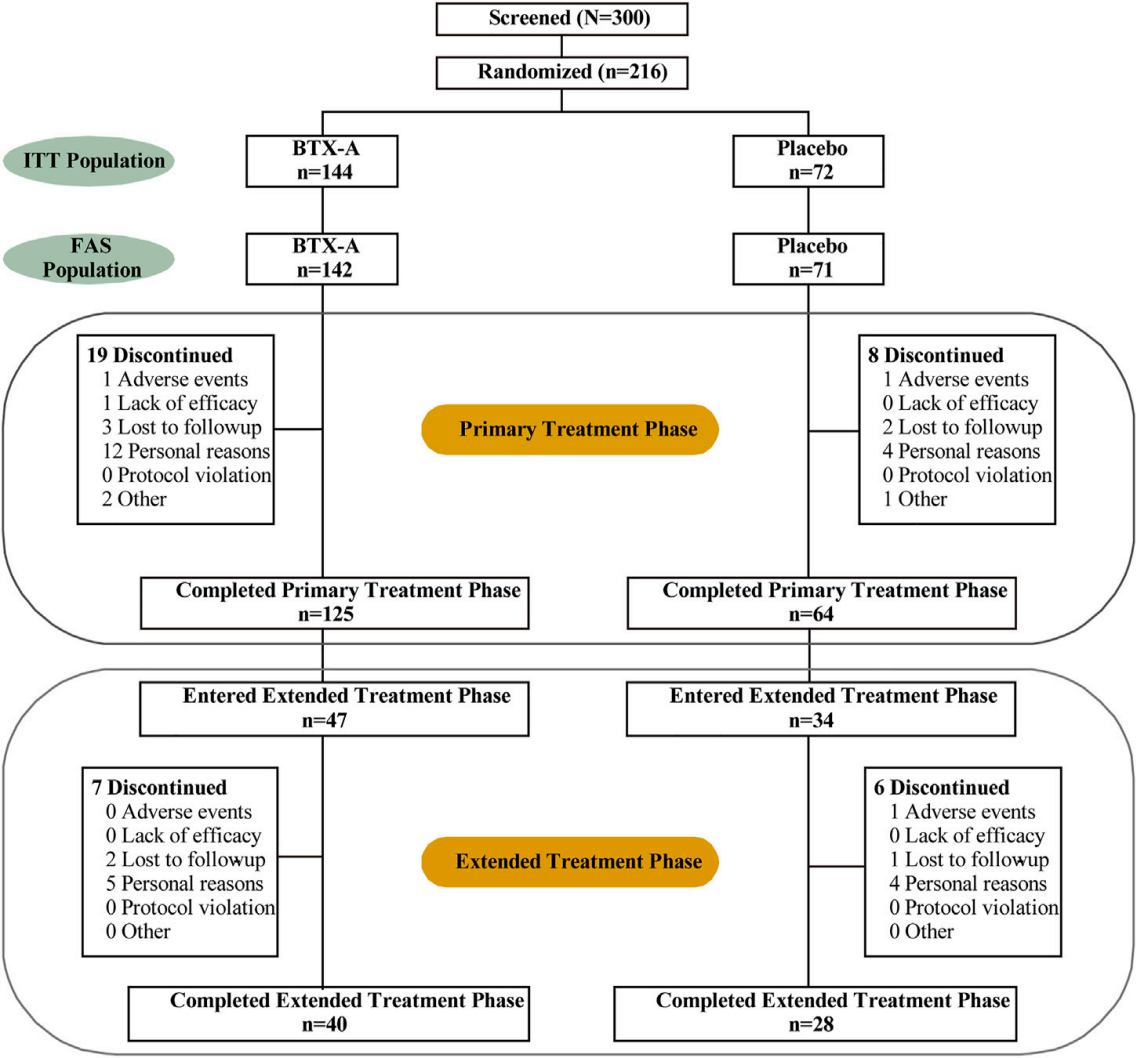
Patient disposition and flow. ITT = intention to treat; FAS = full analysis set.

The ITT (intention-to-treat) populations consisted of 216 randomized patients (144 patients in the BTX-A group and 72 patients in the placebo control group), of whom 189 completed the 12-week study and 27 terminated early (Figure 1). In addition, 34 and 47 patients initially received BTX-A and placebo received retreatment, respectively. The FAS populations included 213 patients, 142 in the BTX-A group and 71 in the placebo group. AEs caused discontinuation in three patients. There was a balanced distribution of baseline characteristics between the treatment groups (Table 1). The duration of OAB was 0.57 (0.12, 1.99) years, and 1.00 (1.00, 2.00) treatment measures were administered in the past 6 months before entry into the study. Inadequate efficacy was the primary cause of failed OAB management. Patients had 14.00 (11.15, 18.85) micturitions per day at baseline.

**TABLE 1:** Baseline demographics and disease characteristics of FAS population.

Characteristic	BTX-A 100U (*n* = 142)	Placebo (*n* = 71)
Age, yr	47.75 ± 14.20	46.39 ± 15.55
Age group, no. (%)		
≥65 years	21 (14.79%)	11 (15.49%)
<65 years	121 (85.21%)	60 (84.51%)
Female, no. (%)	117 (82.39%)	61 (85.92%)
Weight, kg	59.00 (53.00, 67.00)	57.00 (51.00, 65.00)
Height, cm	160.00 (156.00, 164.00)	160.00 (155.00, 164.00)
Duration of OAB, yr	0.51 (0.08, 1.89)	0.60 (0.18, 2.23)
Prior anticholinergic use		
Patients taking combined anticholinergic drugs during the trial	15 (10.56%)	7 (9.86%)
Treatments taken in the past 6 months	1.00 (1.00, 2.00)	1.00 (1.00, 2.00)
Daily micturition episodes, no.	14.70 (11.30, 19.30)	13.30 (11.00, 16.70)
Micturition volume per time, ml	92.25 (64.30, 142.70)	97.95 (67.10, 159.10)
Daily UI episodes, no.	0.00 (0.00, 0.30)	0.00 (0.00, 0.70)
Daily urgency episodes, no.	12.85 (10.00, 18.30)	13.15 (10.00, 16.30)
Urgency score	2.83 ± 1.03	2.74 ± 1.11
OABSS	9.00 (7.00, 10.00)	9.00 (7.00, 11.00)
OAB degree (Mild, Moderate, Severe)	7 (4.93%); 115 (80.99%); 20 (14.08%)	6 (8.45%); 54 (76.06%); 11 (15.49%)
QoL score	5.00 (5.00, 6.00)	5.00 (5.00, 6.00)

BTX-A, Botulinum toxin A; OAB, overactive bladder; QoL, quality of life; PVR, postvoid residual; UI, urinary incontinence; OABSS, overactive bladder symptom score.

Values are represented as mean ± SD, or median (interquartile range).

### Primary Efficacy End Point

**FIGURE 2:**
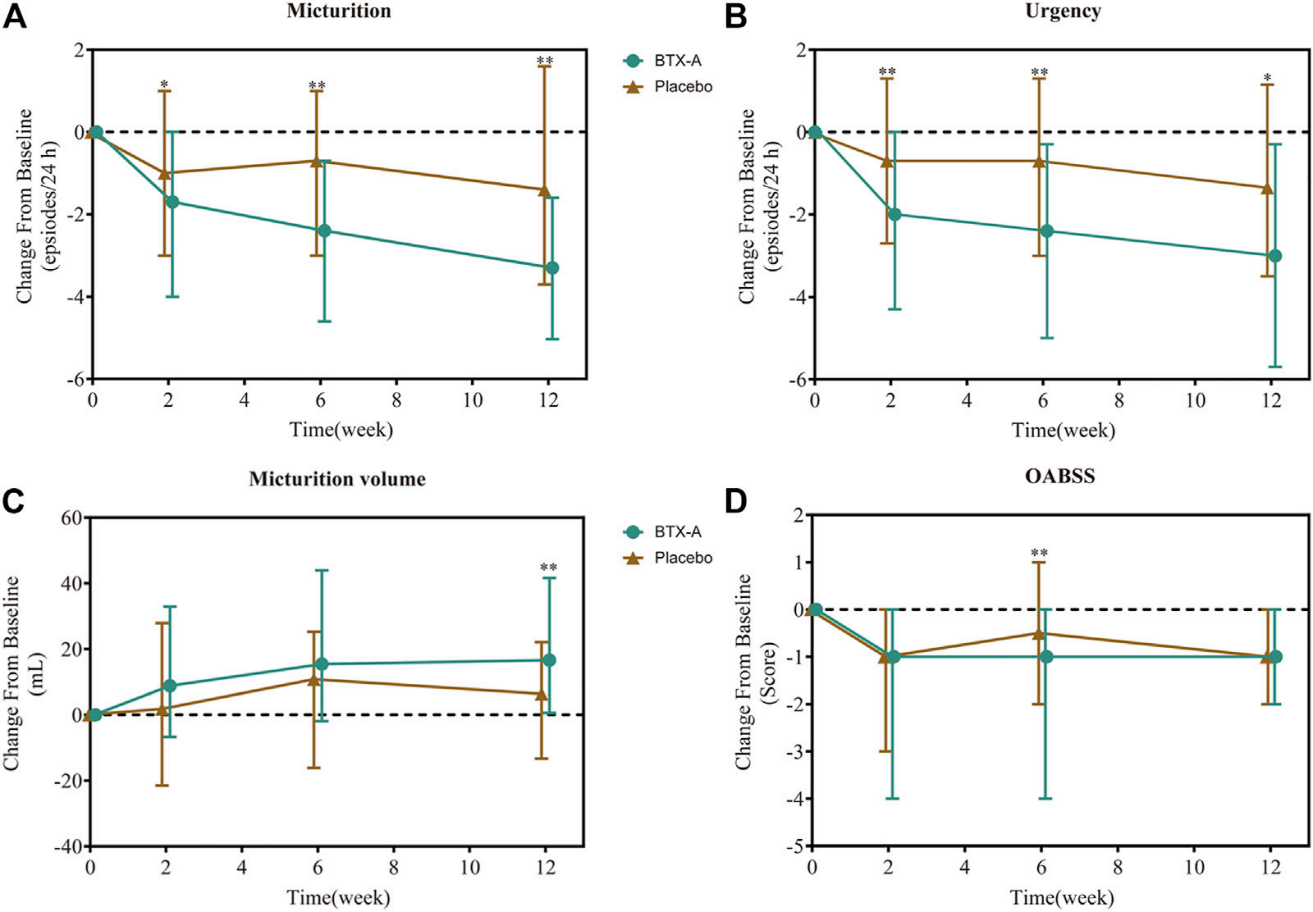
Change from baseline in daily average number of micturition episodes **(A)**, daily average number of urgency episodes **(B)**, average micturition volume per time **(C)**, and OABSS score **(D)**. Values are represented as median (interquartile range). *p < 0.05; **p < 0.01; ***p < 0.001 vs. placebo.

After 6 weeks, all treatment groups demonstrated significant reductions in the average number of micturition episodes per 24 h from baseline, and the difference between the two groups was statistically significant (*p* < 0.001 and *p* = 0.008 respectively). Compared with baseline, the number of micturition episodes per 24 h decreased by 2.40 (0.70, 4.60) times for the BTX-A group and 0.70 (−1.00, 3.00) times for the placebo control group, and the difference between the two groups was statistically significant (*p* = 0.003; Figure 2A). Furthermore, the change rates of average micturition episodes per 24 h from baseline at week 6 in the BTX-A group was significantly greater compared with placebo (16 vs. 8%, *p* = 0.014; Table 2).

**TABLE 2:** Change from baseline in daily average episodes up to post-treatment week 12 in FAS population.

	Mean change from baseline		*p* Value
	BTX-A 100 U, *n* = 142	Placebo, *n* = 71	
Micturition episodes			
Week 2^*^	−1.70 (0.00, −4.00)	−1.00 (1.00, −3.00)	0.050
Week 6^**^	−2.40 (−0.70, −4.60)	−0.70 (1.00, −3.00)	0.003
Week 12^**^	−3.30 (−1.60, −5.03)	−1.40 (1.60, −3.70)	0.008
Micturition volume			
Week 2	8.80 (32.90, −6.70)	1.80 (27.90, −21.50)	0.130
Week 6	15.40 (43.90, −1.90)	10.80 (25.20, −16.10)	0.064
Week 12^**^	16.60 (41.60, 0.60)	6.40 (22.40, −13.30)	0.006
Urgency episodes			
Week 2^**^	−2.00 (0.00, −4.30)	−0.70 (1.30, −2.70)	0.010
Week 6^**^	−2.40 (−0.30, −5.00)	−0.70 (1.30, −3.00)	0.003
Week 12^*^	−3.00 (−0.30, −5.70)	−1.35 (1.15, −3.50)	0.025
UI episodes			
Week 2	0.00 (0.00, 0.00)	0.00 (0.00, 0.00)	0.724
Week 6	0.00 (0.00, 0.00)	0.00 (0.00, −0.30)	0.822
Week 12	0.00 (0.00, 0.00)	0.00 (0.00, 0.15)	0.760
OABSS			
Week 2	−1.00 (0.00, −4.00)	−1.00 (0.00, −3.00)	0.160
Week 6^**^	−1.00 (0.00, −4.00)	−0.50 (1.00, −2.00)	0.003
Week 12	−1.00 (−0.50, −4.00)	−1.00 (0.00, −2.00)	0.062
QoL score			
Week 2	−1.00 (0.00, −2.00)	0.00 (0.00, −1.00)	0.131
Week 6	−1.00 (0.00, −2.00)	−1.00 (0.00, −2.00)	0.238
Week 12	−1.00 (0.00, −2.00)	−0.50 (0.00, −2.00)	0.184
Urgency score			
Week 2	−0.10 (0.10, −0.50)	−0.10 (0.10, −0.30)	0.352
Week 6	−0.20 (0.20, −0.70)	0.00 (0.20, −0.30)	0.076
Week 12	−0.20 (0.20, −0.80)	−0.10 (0.30, −0.70)	0.409

*p < 0.05; **p < 0.01. Values are represented as median (interquartile range).

### Secondary and Other Efficacy End Points

In the first visit at week 2 post-treatment, significant differences in the average number of micturitions as well as urgency episodes were already observed compared with the placebo. The average number of micturitions per 24 h at weeks 2 and 12 decreased by 1.70 (0.00, 4.00) and 3.30 (1.60, 5.03) times for the BTX-A group, 1.00 (−1.00, 3.00) and 1.40 (−1.60, 3.70) times for the placebo control group compared to baseline; the difference between the two groups was statistically significant (*p* = 0.008 and *p* = 0.014, respectively; Figure 2A). Efficacy was also observed in patients retreated with BTX-A. Moreover, BTX-A significantly decreased the number of urgency episodes compared with placebo 2, 6 and 12 weeks after treatment (*p* = 0.010, *p* = 0.003, and *p* = 0.025, respectively). The decreased number of urgency episodes from baseline at week 6 were 2.40 (0.30, 5.00) and 0.70 (−1.30, 3.00) for the BTX-A and placebo groups, respectively (Figure 2B). At week 12, the average micturition volume per time in the BTX-A group was significantly higher than the placebo group (*p* = 0.01; Figure 2C). Significant reductions in the OABSS from baseline were also observed during the primary phase following treatment with BTX-A (*p* = 0.003), with a decreased OABSS score by 1.00 (0.00, 4.00) and 0.50 (−1.00, 2.00) from baseline for the BTX-A and placebo groups at week 6 (Figure 2D). Nevertheless, no significant changes were observed at other follow-up periods. There was, however, no statistically significant difference between the groups in terms of the mean daily urgency score, the QoL score, and the number of UI episodes compared to baseline at each follow-up evaluation (Table 2).

### Safety and Tolerability

AEs were mainly localized in the lower urinary tract. The most common AEs in the BTX-A group, occurred within the first 12 weeks of treatment, as well as over the entire treatment cycle, as follows: increased PVR (19.01%); dysuria (14.79%); and UTI (13.38%; Table 3). UTIs were all uncomplicated with no involvement of the upper urinary tract.

**TABLE 3:** Safety parameters in two consecutive treatment phases in the safety population.

Adverse event	Primary treatment phase	
	BTX-A 100U, *n* = 142	Placebo, *n* = 71
AEs with incidence >2%, no. (%)		
PVR		
≤100 ml	13 (9.16)	3 (4.23)
>100 ml, ≤200 ml	10 (7.04)	0 (0)
>200 ml, ≤300 ml	3 (2.11)	0 (0)
>300 ml	1 (0.70)	0 (0)
Total	27 (19.01)	3 (4.23)
Dysuria	21 (14.79)	6 (8.45)
UTI	19 (13.38)	6 (8.45)
Haematuria	3 (2.11)	1 (1.41)
ALT (Alanine transaminase) elevation	3 (2.11)	0 (0)
	Extended treatment phase	
	BTX-A 100U, *n* = 81	
AEs with incidence >2%, no. (%)		
PVR		
≤100 ml	7 (8.64)	
>100 ml, ≤200 ml	4 (4.94)	
>200 ml, ≤300 ml	0 (0)	
>300 ml	0 (0)	
Total	11 (13.58)	
UTI	6 (7.41)	
Dysuria	6 (7.41)	
Urinary retention	2 (2.47)	

**FIGURE 3:**
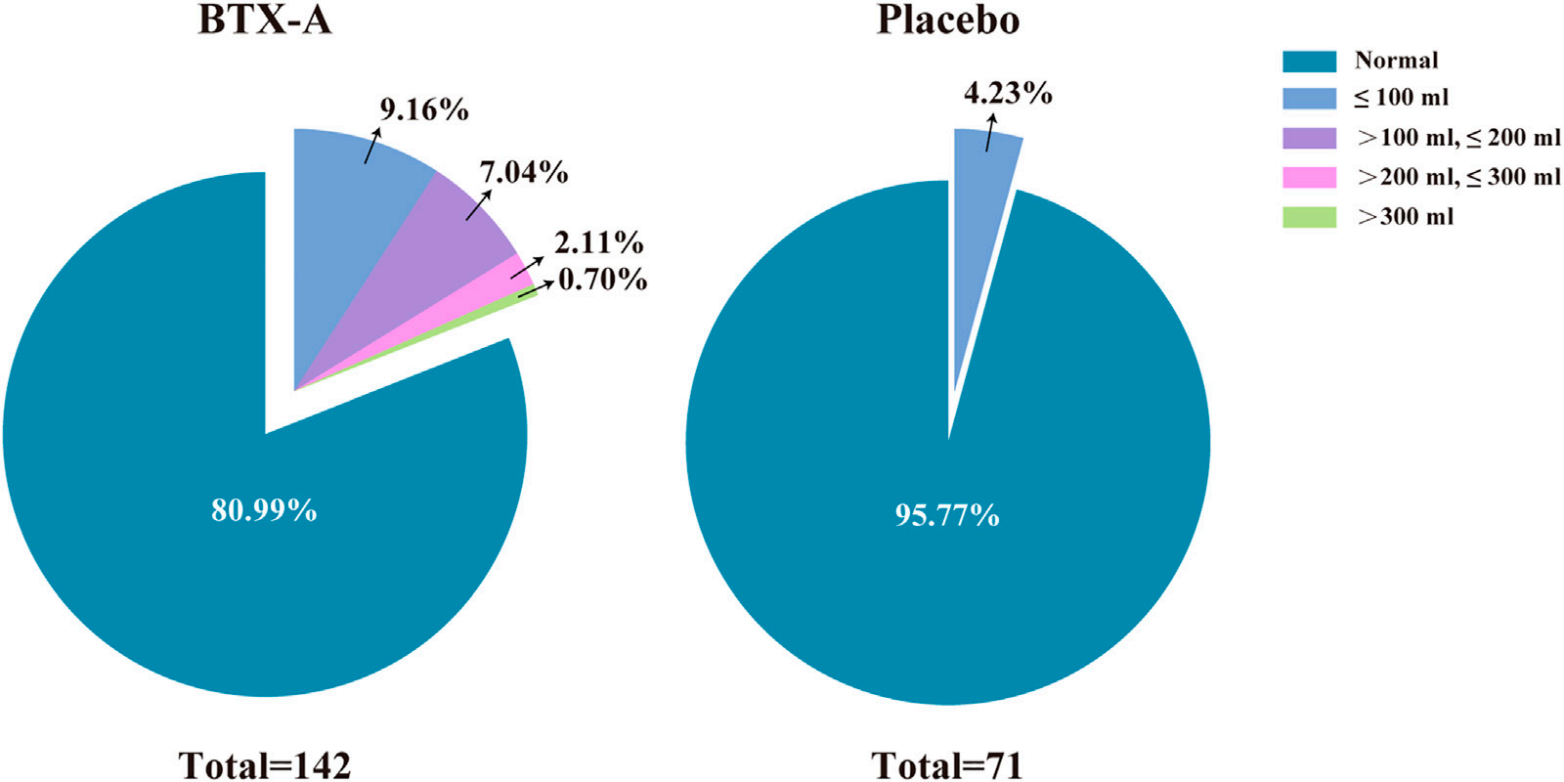
Proportion of safety population with increased PVR during the primary treatment phase.

There were no statistically significant differences in the incidence of AEs between groups during the primary treatment phase, with the exception of PVR (*p* = 0.003). There was one patient (0.7%) who had a PVR >300 ml among the 27 patients (19.01%) in the BTX-A group during the primary treatment phase. The PVR in the placebo group for all 3 patients (4.23%) did not exceed 100 ml during the primary treatment phase. Of the 11 patients (13.58%) with an increased PVR in the extended treatment phase, 7 patients (8.64%) had a urine volume <100 ml. No patients had a PVR >200 ml during the extended treatment phase (Figure 3). There was one patient who required CIC for urinary retention during treatment (Table 3). In other cases, the patients managed their PVR with oral medications or did not require treatment, and the PVR decreased spontaneously over time.

The BTX-A and placebo groups had low discontinuation rates due to AEs. Specifically, 1.41 and 0.70% of patients withdrew during the primary treatment phase, respectively. No deaths occurred in this study.

## Discussion

This study is the first multicenter, randomized, double-blind, placebo-controlled study in Chinese patients with OAB who were inadequately managed (insufficient efficacy or intolerable side effects) with anticholinergic therapy. The results at 6 weeks showed that treatment with Hengli^®^ BTX-A was associated with statistically significant improvement in the primary efficacy end point (number of daily micturition episodes). Patients perceived an overall improvement in their condition, which was clearly supported by the results of the micturition episodes, urgency episodes and OABSS. Results from the present study are comparable to the two phase III trials carried out in the U.S. and Europe, where the mean difference in daily micturition episodes from baseline at week 6 after Botox treatment was −1.96 and −2.40, respectively (Chapple et al., 2013; Nitti et al., 2013), which was in agreement with the mean decrease of −3.28 observed in the present study. OABSS improvements with BTX-A treatment were also similar to the results in a study conducted in Japan, with a mean decrease of −3.4 in the previous trial compared with −1.94 in this trial (Yokoyama et al., 2020). Finally, the results of Hengli^®^ BTX-A in this trial were comparable to other trials that showed Botox treatment had a positive effect on OAB symptoms.

Hengli^®^ BTX-A was well-tolerated in this study. In the BTX-A group there were more AEs and treatment-related AEs that were mainly related to the urinary tract. These factors included an increase in PVR, dysuria, UTI, and urinary retention. A great number of patients in the BTX-A group had a PVR >100 ml that was either managed with medication or ignored; the PVR decreased as a result. CIC was initiated by only one patient which was less than previous studies (Chapple et al., 2013; Nitti et al., 2013; Yokoyama et al., 2020). Most adverse events were mild or moderate in severity. The number of study discontinuations in the BTX-A group was quite low (1.41%). The safety of Hengli^®^ BTX-A in the current study was comparable to Botox in trials conducted in the United States and Europe.

The active pharmaceutical ingredient (API) of Hengli^®^ is crystalline neurotoxin. Like Botox^®^, Xeomin^®^, and Dysport^®^, the neurotoxin in Hengli^®^ is derived from the Hall strain of *C. botulinum* type A and has an identical amino acid sequence. The high-performance liquid chromatography (HPLC) analysis of neurotoxin determined that its molecular mass was 150 kDa, with a heavy chain of 100 kDa and a light chain of 50 kDa. Its chemical purity reached 99.5% (Zhao et al., 2010). Hengli^®^, like other products, is a complex mixture of compounds containing botulinum neurotoxin, complexing proteins, and excipients. In contrast to Xeomin^®^, which only contains the 150 kD neurotoxin, Hengli^®^, Botox^®^, and Dysport^®^ contain the 150 kD neurotoxin as part of a complex with additional proteins which do not participate in the mechanism of action. The SDS-PAGE of Hengli^®^ complex exhibited the following protein bands: neurotoxin (152 kDa), nontoxic non-HA (136 kDa, 120 kDa), hemagglutinin (HA) 70 component (57 kDa, 17 kDa, 15 kDa), HA 33 component (30 and 28 kDa). These basic components are common to all products with BTX-A complex. Besides, Hengli^®^ uses gelatin as the stabilizing protein rather than human serum albumin. Other adjuvant preparations include dextran and sucrose. Comparison of the potency of Hengli^®^, Botox^®^, and Xeomin^®^ shows no difference in their potency labeling (Liang et al., 2017; Dressler, 2020). Hengli^®^ contains about 5 ng of API per vial (100U), so a small amount of protein confers clinical benefit while posing a very low risk of stimulating the immune system. No neutralizing antibody was detected against active BTX-A after four repeated-dose animal studies with Hengli^®^ (Liang et al., 2017).

The clinical efficacy of Hengli^®^ BTX-A may result from its dual action involving both efferent and afferent processes. In the efferent nerves, BTX-A injections temporarily inhibit the contraction of detrusor tissues by blocking the release of acetylcholine through cleavage of SNAP-25 in both the pre- and post-ganglionic nerves (Cruz, 2014). Additionally, BTX-A blocks the release of ATP from purinergic efferent nerves in the detrusor muscle (Lawrence et al., 2010). A BTX-A injection inhibits the conduction of the afferent nerves in the bladder detrusor by decreasing urothelial ATP release and increasing urothelial NO release (Khera et al., 2004; Collins et al., 2013). Moreover, reduction in TRPV1 and P2X3 immunoreactivity in biopsies of patients with OAB after a BTX-A injection is associated with better clinical outcomes (Apostolidis et al., 2005). Overall, BTX-A injections may ameliorate OAB symptoms at multiple levels. Further research could shed light on the comprehensive mechanism underlying BTX-A action.

In contrast to randomized controlled trials that evaluate Botox^®^ or other BTX-A products in OAB patients, micturition episodes were chosen as the primary endpoint to evaluate efficacy in this study. This setting was based on the results of an epidemiological study of OAB in China, in which 70% of Chinese OAB patients present without UI (OAB-dry) (Wang et al., 2011). In accordance with this conclusion, our data also showed that most Chinese OAB patients rarely suffer from urinary incontinence (Table2). Furthermore, our study showed Hengli^®^ is comparable to Botox^®^ when it comes to relieving frequency in patients with OAB. Additionally, due to the double-blinded nature of this study, researchers and patients were both blinded to treatment, so an ethically sound method was used. Therefore, patients were not required to stop taking anticholinergic medications throughout the study, but rather to maintain the same dosage as before enrollment.

It is worth investigating whether gender factors affect the responses to BTX-A treatment in overactive bladder patients, as there is a gender factor in OAB pathophysiology. In this study, 82.39% of patients in BTX-A group and 85.92% of patients in placebo group were females. However, when comparing the safety and efficacy of botulinum toxin after using sex grouping, there was no significant difference. Our results may be influenced by the large difference in the number of patients between males and females, as we did not restrict recruitment to certain genders. Another male-female cohort study in a larger patient population would provide insights into differences in responses to botulinum toxin between genders.

One limitation of the study was that comparisons with placebo were not feasible after week 12, because patients were able to request and receive retreatment beyond that point. In an extended study phase, repeated treatment with Hengli^®^ BTX-A are being evaluated to determine the efficacy over the long term. Secondly, no statistically significant differences were found between the groups in terms of the number of UI episodes compared to baseline at each follow-up evaluation, which was the primary efficacy endpoint of Botox trials conducted in the U.S. and Europe. Consistent with prior epidemiologic studies, there were fewer UI episodes among the 2 groups at baseline (Wang et al., 2011). As a result, fewer UI episodes may result in an insignificant difference between groups. Moreover, no significant difference was observed in QoL scores between the Hengli^®^ and the placebo group. As the International Prostate Symptom Score-QoL Subscore questionnaire was used to evaluate QoL, which consists of one question with a score between 0 and 6. In this questionnaire one highly subjective question was asked, and the OAB population tends to have extremely high expectations of efficacy, thus a statistically significant score may not be obtained.

## Conclusion

Hengli^®^ BTX-A treatment resulted in significant improvement of OAB symptoms compared with placebo in Chinese OAB patients who were poorly managed by anticholinergic therapy. The participants in this study reported improvements in their condition. In Chinese OAB patients, Hengli^®^ BTX-A was well-tolerated, with a low incidence of PVR elevation requiring CIC. In this study we showed that Hengli^®^ BTX-A is an effective treatment option for Chinese patients with OAB in whom prior anticholinergic therapy failed to manage their condition adequately.

## Data Availability

The original contributions presented in the study are included in the article/Supplementary Material, further inquiries can be directed to the corresponding author.
